# Challenges and Benefits of an Internet-Based Intervention With a Peer Support Component for Older Adults With Depression: Qualitative Analysis of Textual Data

**DOI:** 10.2196/17586

**Published:** 2020-06-16

**Authors:** Annie T Chen, Krystal Slattery, Kathryn N Tomasino, Caryn Kseniya Rubanovich, Leland R Bardsley, David C Mohr

**Affiliations:** 1 Department of Biomedical Informatics and Medical Education University of Washington School of Medicine Seattle, WA United States; 2 Department of Preventive Medicine Center for Behavioral Intervention Technologies Northwestern University Feinberg School of Medicine Chicago, IL United States; 3 Division of Gastroenterology & Hepatology Department of Medicine Northwestern University Feinberg School of Medicine Chicago, IL United States; 4 San Diego State University/University of California San Diego Joint Doctoral Program in Clinical Psychology San Diego, CA United States

**Keywords:** aged, depression, internet, peer group, social support, qualitative research

## Abstract

**Background:**

Technological interventions provide many opportunities for improving the health and quality of life of older adults. However, interaction with new technologies can also cause frustration. Although these themes have been explored in extant research, much remains to be learned with regard to how the challenges of aging and technology use and the experiences of participating in a social and learning environment are interrelated.

**Objective:**

This study aimed to perform a qualitative analysis of data collected from MoodTech, a pilot study of an internet-based intervention with a peer support component for older adults with symptoms of depression, to better understand the participants’ experience of using technological interventions, including the challenges and benefits that they experienced over the course of these interventions.

**Methods:**

We employed an inductive qualitative analysis method based on grounded theory methodology and interpretative phenomenological analysis to analyze participant textual data. These textual data were of 3 main types: (1) assignments in which participants challenged their negative thoughts, (2) status updates, and (3) comments in the peer support component of the intervention.

**Results:**

We have presented the results through 3 main themes: (1) the challenges of aging as seen through the participants’ comments, (2) the difficulties experienced by the participants in using MoodTech, and (3) the benefits they derived from participating.

**Conclusions:**

This paper offers several contributions concerning study participants’ experiences with internet-based cognitive behavioral therapy (iCBT) interventions with a peer support component and design considerations for developing complex technological interventions that support the challenges participants experience due to aging and cognitive difficulties. First, technical issues encountered by older adults within the context of the intervention can interact with and exacerbate the insecurities they experience in life, and it is important to consider how intervention components might be designed to mitigate these issues. Second, peer support can be employed as a mechanism to facilitate communication, support, and collaborative problem solving among participants in an intervention. The insights from this paper can inform the design of iCBT interventions for older adults.

## Introduction

### Background

Depression is a psychiatric disorder that affects a significant proportion of older adults [1,2] and can have various negative consequences, including decreased quality of life, increased disability, and increased risk of mortality [3]. In addition, depression can often go undetected or untreated [2,4]. The treatments for depression in older adults include antidepressants, behavioral treatments, cognitive behavioral therapy (CBT), cognitive bibliotherapy, problem-solving therapy, psychotherapy, and life review/reminiscence therapy [2,5,6]. In recent years, there has been interest in internet-based CBT (iCBT) for reducing depressive symptoms in older adults; however, the number, quality, and heterogeneity of extant research suggest the need for additional research to evaluate the efficacy of iCBT interventions [7].

There has also been recognition of the negative effects of social isolation among older adults, including poorer cognitive functioning, impaired sleep and daytime dysfunction, reductions in physical activity, impaired mental health, and the potential utility of technology for alleviating loneliness and social isolation [8,9]. Although studies of technological interventions for depression and loneliness among older adults are still limited, extant research suggests that these interventions can reduce the symptoms of anxiety and depression [10,11] and are helpful for the management of loneliness [8,12,13].

However, older adults may experience difficulties in learning new technologies, and it is important to provide support during the process of learning to use them [14]. Although digital technologies could be a vehicle for empowerment by facilitating greater engagement in hobbies, social support, and everyday tasks, they may also be a form of disempowerment if they are viewed with apprehension, and the inability to use them can lead to social isolation [15]. Moreover, the adoption of technology use can be mediated by cognitive abilities, computer self-efficacy, and computer anxiety [16].

Nevertheless, previous research has suggested that older adults perceive that the benefits of using these technologies may outweigh the costs [17] and that engagement with internet and digital technologies can lead to improved depression symptomatology and psychological well-being [18,19]. In recent years, there has been increased interest in the factors that influence engagement with social networking websites among older adults [20]. For example, some older adults use Facebook to connect with family, and previous research has reported that older adults who were Facebook users scored higher on assessments of social satisfaction than those who were not [21]. Taken together, the extant literature suggests that new technologies and web-based social environments may benefit older adults, but the implementation of these environments is critical.

### Objectives

Although prior research on digital health interventions has examined the effect of the peer support component using quantitative measures [11,22,23], there is great potential to employ qualitative data to better understand participants’ experiences of peer support interventions and iCBT interventions for older adults. In this study, we performed a qualitative secondary analysis of data from MoodTech, an internet-based intervention for depression for adults aged 65 years and above [11]. This intervention included didactic content (in the form of lessons), interactive skills–based tools, and peer support features. The primary analysis focused on quantitative analyses of engagement metrics (participant log-ins and minutes); usability; and mental health outcomes, including depression and anxiety, and reported a reduction in depression among participants compared with those waitlisted [11].

In this study, we focused on the textual data that participants produced, either as part of the activities they engaged in along with the didactic content or in the peer-support component of the website, to understand the participants’ experiences of the intervention and develop recommendations for the design of internet-based interventions for older adults. We describe three themes, namely, challenges experienced by older adults in life, difficulties experienced in the intervention, and the benefits of the online social space, using the first theme of life challenges to provide context for the other two themes.

## Methods

In the Methods section, we introduce the intervention, explain the data that are the focus of the analysis, and then describe the data analysis method.

### MoodTech

**Figure 1:**
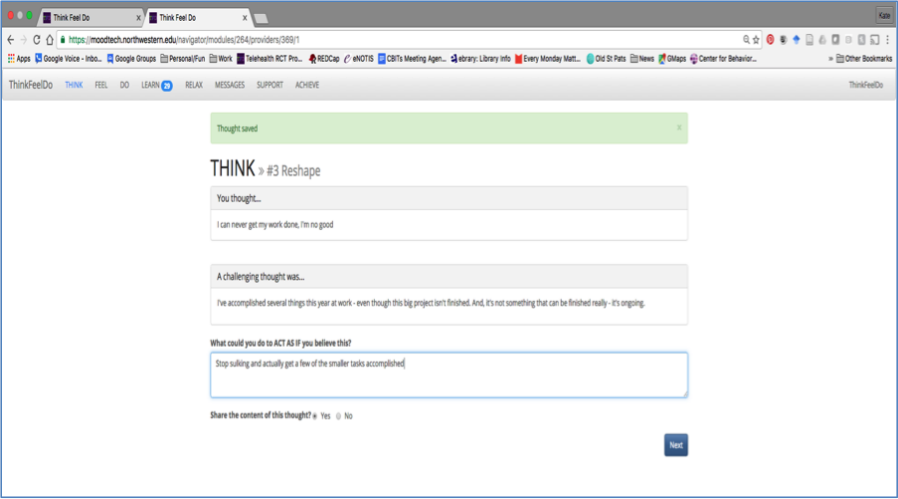
Screenshot of harmful thoughts activity. Participants enter thoughts and create a challenging thought and action in response. The thoughts can be private or shared publicly with the peer support group, allowing peer and coach comments.

The MoodTech study was designed to evaluate the feasibility and acceptability of an online intervention for depression based on CBT principles, developed for participants aged 65 years and above [11]. MoodTech was built on the ThinkFeelDo platform [24] and focused on five core skills: (1) cognitive restructuring (think), (2) mood and emotion monitoring (feel), (3) behavioral activation (do), (4) relaxation/mindfulness (relax), and (5) goal setting (achieve). Activities were an aspect of the *do* tool. Within the tool, participants were able to list activities completed in the last few days. Participants could plan activities to boost their mood, provide an optional prospective due date for completion, and report completion. The *think* tool asked the users to record their negative thoughts, categorize the type of cognitive distortion (eg, magnifying, minimizing and tunnel vision), reframe the thought constructively, and create an action plan (Figure 1).

**Figure 2:**
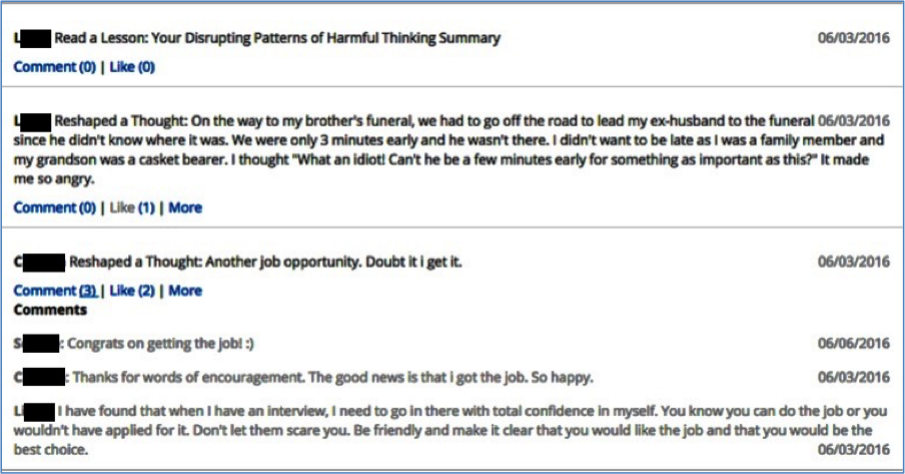
Screenshot of the social peer support feature displaying a portion of social media feed where a negative thought shared publicly had 3 comments and 2 likes.

The peer support version of the intervention also featured an online social environment in which the participants could interact with one another (Figure 2). This included an activity feed that automatically posted the participants’ actions, including lessons read, goals set, and relaxation exercises completed. The participants could share status updates and comment on items shared by others. They also had the option of sharing thoughts they entered as part of the *think* tool and activities they completed or scheduled in the *do* tool.

The study comprised 3 treatment arms, each 8 weeks in length: 1 group received the online intervention without peer support (individual internet-based intervention), another group (divided into 2 cohorts) received the online intervention with peer support (internet-based intervention with peer support), and the final group was a waitlist control group that was evaluated with no treatment during the course of intervention before being given access to the individual internet-based intervention.

Participants were primarily recruited through clinical research registries as well as online and community advertisements and clinic referrals. To participate, individuals needed to be aged ≥65 years; to have elevated depressive symptoms at screening (score of ≥8 on the patient health questionnaire-8 or a score of >7 on the geriatric depression scale-15); to be able to read and speak English; and to have basic internet skills and access to the internet, a telephone, and a valid email address. The exclusion criteria included current engagement or planned engagement in psychotherapy, psychiatric diagnoses for which participation could be inappropriate (eg, psychotic disorder), or cognitive impairment [11].

### Data Set

The MoodTech data set comprises demographics, usage, and outcomes data collected before and during the MoodTech intervention. We report the descriptive statistics and feature usage statistics for the sample.

During the intervention, participants produced textual data incidentally through their interaction with the system. First, participants produced textual data through individual activities, such as the goals they set for themselves, and their work on challenging negative thoughts. Status updates were part of the activity feed, allowing users to post unsolicited statements to their peers on any topic. Comments and *likes* could be made in response to any material in the activity feed, including thoughts or goals that were publicly shared, user profiles, or status updates, and were visible to all participants in the intervention. Comments were the only means of direct text communication among the participants and often would resemble conversation, with multiple comments back and forth attached to an original post to the feed. The participants were also able to *nudge* (a social action resembling a physical *nudge*, which carries no verbal communication) other participants to promote engagement. The intervention also included a *messages* section, where participants could interact privately with their coach. The coach could also comment on the participants’ activity on the feed.

In this study, we focused on textual data from thought activities, status updates, and comments as these tended to be longer and richer, thus providing more insight into participants’ experiences. The textual data were aggregated into documents by participant ID and textual data type (activities, thoughts, status posts, and comments) for analysis, such that each document represented the textual expression of 1 participant for a given data type. For example, all status posts by ID 1523 would have been aggregated into a single document. To provide context for analysis of comments, the original status post or item (eg, harmful thought) that elicited the comment was also included in these documents. We focused on the text generated as part of the *think* section or in the social space (ie, status updates and comments), which provided the richest insight into participants’ experiences of the MoodTech platform and their efforts to practice the skills taught in the intervention.

### Qualitative Data Analysis

Our analytic method was informed by two qualitative approaches: interpretative phenomenological analysis (IPA) [25] and grounded theory [26-28]. At the outset, the perspective with which we viewed the data was influenced by IPA, a method that places emphasis on how individuals make sense of their world, positing a close connection between what a person says or writes and his or her cognitions [25,29]. IPA is widely used in health-related studies with a focus on patient experience [29-31]. In this study, we adopted this perspective in code development, endeavoring to create codes that illustrated participants’ experiences of life and the intervention.

Our analytic procedure was based on grounded theory methodology. In grounded theory, researchers code the data line by line [26]. The codes are refined through a process involving constant comparisons and constant questioning. We identified concepts of interest, namely, health, depression, aging, technology, and participation in the social networking component of the intervention, and 1 author (KS) performed line-by-line coding, creating codes that were related to one or more of these topics. Then, 2 authors (KS and AC) worked together to iteratively refine and merge codes and to create a hierarchical code structure of themes and subthemes. After KS developed the initial codes and hierarchical structure of themes, AC reviewed the codes and structure and suggested revisions, which were then reviewed by KS. The 2 authors agreed upon the final set of codes and hierarchical code structure. The codes were compared for the same participant and across participants to ensure consistency of the coding scheme, focusing on the reduction of conceptual overlap and redundancy. The two authors consulted with a clinical expert (KT) to ensure that the interpretation of the themes was consistent with the clinical knowledge of depression in older adults and with the design of the MoodTech intervention. The data analysis was performed using the qualitative data analysis software, Atlas.ti (Cleverbridge, Inc) [32].

Although we initially included activities (data from the *do* tool) in our analysis, we found that they were usually short phrases or sentences indicating what the participants planned or had already done (eg, *ate supper,* ID 1568) and thus uninformative concerning the participants’ inner mental state. Therefore, we did not report the themes from the activities in this paper. In selecting the themes to report and elaborate upon, we considered the guidelines provided by Braun and Clarke [33], with frequency and conceptual relatedness of the codes to one another being key considerations. In this paper, we focused on participants’ life challenges, difficulties with the program, and benefits of the program. In our selection of quotes, we endeavored to represent the diversity of voices among the sample.

The aims of our investigation were to qualitatively understand how the participants experienced the intervention material and the peer support component. With respect to the first goal, all participants, regardless of treatment arm, received the same intervention materials; thus, we analyzed the available data from all treatment arms together. With regard to the second goal of analyzing participants’ experiences with the peer support component, we only used data from the one arm that received this component, the internet-based intervention with peer support.

## Results

### Data Set

#### Sample

The MoodTech study included 47 participants. Two of the original participants (1 in the individual internet-based intervention and the other in the wait list control) were not included in this data set. One participant in the individual internet-based intervention group dropped out of the study before using the intervention, and one of the 12 participants in the wait list control decided not to use the intervention after the waiting period. Most of the participants were female, white, and retired. Overall, the sample was highly educated, with most participants having a 2-year college degree or above. The summary statistics for the sample are shown in Table 1.

**Table 1:** Participant characteristics.

Participant characteristic		Individual internet-based intervention		Internet-based intervention with peer support		Wait list control	
Age (years), mean (SD)		69.3 (3.5)		69.5 (4.3)		70.2 (4.9)	
**Sex, n**							
	Female		7		16		7
	Male		4		7		4
**Ethnicity, n**							
	Not Hispanic or Latino		11		22		11
	Hispanic or Latino		0		1		0
**Race, n**							
	Black or African American		0		1		0
	White		10		19		11
	More than 1 race		1		2		0
	Declined to report		0		1		0
**Marital status, n**							
	No partner		7		16		4
	Partner		4		7		7
**Education, n**							
	Some college		0		5		1
	2-year college (Associate’s degree)		0		5		2
	4-year college (Bachelor’s degree)		3		6		4
	Master’s degree		5		5		2
	Doctoral degree (PhD, MD, and JD)		3		2		2
**Employment status, n**							
	Employed		2		3		3
	Retired		9		18		7
	Other		0		2		1

#### Feature Usage

Although most participants engaged with the features of the application they had access to, there was a great deal of variability in the extent to which the features were used (Table 2). A total of 43 out of 45 participants produced text data of at least one type. In the treatment arm with peer support, less than half of the individuals authored status updates, but almost all participants commented on at least one status post. Similarly, with nonverbal social interactions, almost all participants liked an item at least once, but only about half of the participants used the nudges. As the SDs indicate, there was substantial variability in the extent to which the participants used the features of the intervention.

**Table 2:** Engagement metrics for the skills and peer support components of the MoodTech intervention.

Feature used		Individual internet-based intervention (n=11)		Internet-based intervention with peer support (n=23)			Wait list control (n=11)		
		Participants, n	Participants, mean (SD)		Participants, n	Participants, mean (SD)		Participants, n	Participants, mean (SD)
Thoughts		9	9.1 (4.5)		18	11.9 (11.1)		9	10.1 (5.3)
Messages		11	11.6 (5.8)		21	9.3 (7.2)		11	10.4 (6.1)
**Social interactions: verbal**									
	Status posts	N/A^a^	N/A		10	19.7 (16.7)		N/A	N/A
	Comments	N/A	N/A		19	20.3 (23.6)		N/A	N/A
**Social interactions: nonverbal**									
	Likes	N/A	N/A		19	25.7 (52)		N/A	N/A
	Nudges	N/A	N/A		9	5.7 (8.2)		N/A	N/A

^a^N/A: not applicable.

#### Textual Data

Our analysis focused on the textual data produced as a part of the *think* and *do* sections of the intervention and the social space. The activities and harmful thoughts features of MoodTech were available to all 45 participants, whereas status updates and comments were only available to participants in the peer support arm (n=23). Table 3 depicts how the text data were divided into documents, with each document pertaining to a unique participant-data type pairing, and imported into Atlas.ti.

**Table 3:** Textual data used in qualitative data analysis.

Arm	Participants, n	Intervention features (available to all)		Peer support component features		Total documents, n
		Activities	Thoughts	Status post	Comment	
Individual internet-based intervention	11	3	11	N/A^a^	N/A	14
Internet-based intervention with peer support	23	15	20	10	19	64
Wait list control	11	1	9	N/A	N/A	10
Total	45	19	40	10	19	88

^a^N/A: not applicable.

### Qualitative Data Analysis

#### Life Challenges

As part of the didactic component of the MoodTech intervention, participants learned how to reframe and reduce harmful thoughts. We analyzed these harmful thoughts to help us understand the challenges that participants face in life, to which they endeavored to apply the skills that they were learning. We describe these life challenges to provide context and to better understand the difficulties and benefits the participants experienced, both with the MoodTech intervention and with skill application.

#### Health Concerns

Participants in the MoodTech intervention expressed many challenges, including health-related problems such as chronic pain, sleep, weight, physical ability, and potentially negative results from medical tests. These often left them with a sense of regret and hopelessness:

I have feelings of regret and grief over my health and often wish it would speed up and not take so long until it’s over. The pain levels are extremely challenging.

ID1607, harmful thought

When I’m in pain, I feel as though it’s going to last forever, that I’ll never get better...

ID1816, harmful thought

#### Other Challenges

Other than health-related challenges, participants also experienced personal challenges. These could be intertwined with their recollections of the past and concerns about the future. Family played a prominent role. Participants worried about the welfare of their children and their parents, were fearful of losing loved ones, and wrote of tensions they experienced interacting with other family members. They were also concerned about financial status, professional status, and legacy:

I was not a good mother, my kids had to do without so much and work harder.

ID1651, harmful thought

I am afraid that I will not have enough money.

ID1596, harmful thought

Participants also had concerns about how they were perhaps perceived by others. There were various reasons for this, including feeling self-conscious about physical appearance, but some participants were also concerned about how they were perceived in terms of intellect. Participants expressed concern about cognitive difficulties and memory issues:

I can't get my thoughts together; I'm getting worse.

ID1885, harmful thought

I wish my memory was as it once was. I am ALWAYS forgetting names, words, and where I put things as three examples. I feel embarrassed and diminished in the eyes of my family.

ID1838, harmful thought

#### Difficulties That Participants Experienced in the Intervention

##### Technical Difficulties Leading to Self-Doubts

Participants experienced various difficulties in working with the MoodTech intervention. First, in terms of the technology, participants often encountered issues with the system, such as creating a text entry, not having the entry saved, and then having to reenter it. These issues led to confusion and frustration, which were accompanied by the participants questioning their memory and abilities:

The program confuses me and adds to my litany of reasons I think I am a dunce!

ID1523, comment

I think the software challenges on site make several of us feel like dummies and that is no help to alleviate depression.

ID1607, harmful thought

##### Being Overwhelmed by the Pace of the Intervention

Participants felt overwhelmed by the amount of work they perceived that they had as well as the pace of the intervention:

How will I ever get all of this work done in just 7 weeks that I have left?

ID1599, harmful thought

What's the matter with me that I have a hard time keeping up with the lessons in this program and not working on them as much as I should

ID1781, harmful thought

Anyone else finding this program overwhelming. Todays lesson is good but difficult.

ID1596, status post

I'm feeling overwhelmed too. The weekly/daily pressures are too fast-paced for me. I suppose the program needs a schedule but it doesn't take into account the actual pace and work of being depressed. I feel behind too.

ID1550, comment

The problems that participants experienced with the program compounded insecurities and anxieties that participants already had, which led to an increased sense of pressure and frustration.

#### The Benefits of Social Space

Despite the difficulties that the participants experienced with MoodTech, they also experienced positive interactions in the peer support space. These included affirmation and encouragement from peers and coaches, collaborative efforts at problem solving, and positive affective experiences from the social experience of the website.

##### Empathy and Encouragement

Participants expressed empathy and provided encouragement to one another for the difficulties they experienced in life:

This is just a temporary setback (having to keep off your feet for most of the week). You can do it (lost weight) – don’t give up trying. Mind over matter!

ID1816, comment

Looks like you’re on top of the problem and are looking ahead of what comes next.

ID1813, comment

##### Appreciation and Affirmation

At the outset, participants expressed appreciation for and affirmed the contributions of others on the website:

This is a very good insight, [ID 1607]. Although I have not posted very frequently, I feel that you have been very courageous and open during this study.

ID1596, comment

Dear [ID 1781], I have been thinking about your post since last week but couldn’t put my feelings into words. Just wanted you to know that I admire your honesty and the courage that it must have taken to share this post.

ID1760, comment

##### Collaborative Problem Solving

Participants also engaged in collaborative discussions about the problems they were trying to address as part of the MoodTech intervention. For example, in the following exchange, 2 participants discussed the nature of their tendency to worry:

I worry too much – guess I feel that by worrying about all the contingencies, I can somehow control one or two of them. Anyone else have that problem...

ID1816, status post

I also struggle with worrying a lot. [ID 1816] ...I think you nailed it when you said that the reason is that perhaps by worrying, you can control one or two of them...

ID1723, comment

Participants also helped one another figure out how to utilize the website. For example, several participants were confused about how to go from the email notifications of a new comment on their post to the comments themselves. They were able to explain to one another in the social space how to navigate to the particular content that they wanted:

Until I figured out to go to my profile page & scroll down, I'd get the email saying someone commented to me then I'd go to the main page & scroll down through ALL comments until I found mine.

ID1550, comment

I just got a couple of notes that someone “”LIKED“” a couple of my comments. But it's hard to figure out who liked what -- can't I get a link directly to what was liked, so I can acknowledge it?

ID1816, status post

It was confusing to me at first, also. Scroll down all the things that you posted on the Feed. When you find one with a number in parenthesis behind the comment or like, click on the number. That will take you to the comment and who liked it or made a comment. HOpe this helps.

ID1723, comment

## Discussion

### Principal Findings

In this paper, we present a qualitative analysis of textual data collected from MoodTech, a pilot study of an internet-based intervention with a peer support component for older adults with symptoms of depression. We analyzed 3 main types of data from the intervention: harmful thoughts, status updates, and comments. We present the results through 3 main themes: (1) challenges that participants experienced in life, (2) difficulties that participants experienced during the course of the intervention, and (3) the benefits that they derived from participating in the social space. The thoughts section of the data (individual) provided the most insight into difficulties experienced in the intervention, whereas the status post and comment sections (peer support) provided insight into the challenges and difficulties experienced as well as the benefits of having a peer support component in an intervention.

At the outset, participants shared various life challenges that they experienced, including health concerns, self-consciousness, recollections of the past, and anxiety about the future. These concerns provide context for the difficulties that the participants experienced as they engaged in the intervention activities, including being overwhelmed by the pace of the intervention and technical issues. The difficulties they experienced with the intervention fueled the insecurities they were experiencing in life. However, the peer support component appeared to benefit the participants by providing a medium for empathy, encouragement, appreciation, and affirmation, and collaborative problem solving.

The life challenges mentioned by the participants in this study went beyond health-related challenges to include other challenges in which the effects of time and aging were clearly related. This characterization of life challenge resonates with extant literature on aging, which has noted that there is a difference between objective (health-related) and subjective perceptions of health [34]; people with multiple chronic conditions may rate themselves as healthy and active, whereas those with few or no conditions may rate their health more poorly, because of psychological and social factors, coping mechanisms, and resilience. The data collected from the social space can provide insights for researchers into the challenges experienced by the participants in the aging process as well as about how these challenges may affect the participants’ experiences of the intervention.

### Social Space for Information Sharing and Mutual Encouragement

The MoodTech intervention not only offered participants an opportunity to practice the skills that they were learning individually but also with the option of sharing them with others. Although the participants in this study experienced frustration and confusion due to technical difficulties, the participants’ written utterances also suggested that the ability to interact in the social space was a positive experience and that they valued the support and camaraderie of others.

The interactions among the participants in the social space showed that the participants were motivated to help one another with technical issues as well as with the difficulties they experienced in life or with the intervention content. The content and shared understanding from peer interactions made it possible to brainstorm coping strategies or at least take comfort in knowing that others shared their experiences. With regard to the technology, participants understood the frustrations that others were experiencing as well as the insecurities that were perhaps triggered by age-related factors and were quick to offer advice and workarounds to reduce others’ struggles. The findings thus illustrate that a social networking component within the context of a cognitive behavioral intervention can perhaps provide a synergistic contribution to the intervention and enrich and deepen the learning that participants are engaged in with the educational modules.

One question that might arise from this is if there was an optimal level of usage of the features. There was extensive variability in the extent to which the study features were used. Although not all participants posted status updates, almost all participants commented at least once and liked the items shared by others. Participants commented on others’ thoughts and behaviors and expressed admiration for the courage of others who shared their experiences.

Only about half of the participants posted status updates and nudges, and we can only speculate about the causes. Although the reasons for lower usage of these features are unclear, perhaps posting a status update required more courage or effort than simply commenting on the posts of others. With regard to nudges, some possible reasons might be that the meaning of a nudge was less intuitive to participants or more personal than they wanted it to be.

Overall, these findings are consistent with prior literature, which reported that sharing of experiences on online support groups can lead to improved sentiment [35] and patient empowerment [36]. Of particular interest perhaps are contagion effects resulting from participation in a group discussion. As described in the Results, we observed various positive effects of group interactions such as being inspired by the actions of others and resonance in terms of shared experiences leading to subsequent sharing of similar experiences. In some cases, the shared experiences were related to difficulties with the intervention. One participant expressing feelings of frustration with the program can lead to others sharing similar feelings. However, the expression of negative sentiments is not necessarily undesirable. Expressing frustration gives participants a chance to release these tensions and frustrations so that they can be overcome, and, in this intervention, it also led to collaborative problem solving.

In this study, some participants were clearly more active than others, and participants also differed in terms of the nature of content they contributed. Previous research has found that members of online communities play different roles [37]. Previous research investigating lurkers (those who read but do not post) and posters has reported that posters tend to report having their expectations met more often than lurkers [38], those who received emotional support in an online support group were less likely to drop out [39], and online support group members provide different forms of support to one another [40].

On the basis of the comments made in the social space, we suspect that some participants in this study did find the content of others useful even if they did not contribute content themselves and that many perhaps had respect for those who had the courage to do so. It is likely that some participants, whether they contributed or not, benefited from the social space, in terms of both informational and social interactions. However, more research is needed to better understand how different forms of engagement with the social space may be associated with health-related outcomes.

### Design Implications

Participants in this study experienced a substantial amount of frustration due to technical issues. In some but not all cases, this appeared to be exacerbated by other problems they experienced, such as cognitive issues. The problems they experienced led to uncertainty about if they or the system were the cause of the problem. Although the technical difficulties faced by the participants were those that could occur with any system, considering them in the context of age-related factors is critical for delivering effective treatment.

The idea that technological interventions for older adults should consider age-related factors is not new. Prior research, for example, has recommended that technology-based training for older adults should be well-structured, provide feedback and adaptive guidance, include metacognitive prompts, include a user interface that is simple and consistent, and take into consideration extant knowledge of cognitive load [41]. This can be difficult for complex multicomponent educational interventions.

This study provides insights into the design strategies for minimizing confusion and frustration on the part of older adults in the design of educational interventions. At the outset, one of the problems we observed was that participants felt mounting pressure due to the perception that they could not finish their assignments. With MoodTech, a potential alternative design might be to present personalized educational objectives to participants, paced to suit the needs and goals of the participants over time. For example, participants could be told that MoodTech would provide assignments that depended on how they were doing, in a manner similar to how an adaptive system would work. This way, participants would not feel despair at not being able to finish the modules. This might be more difficult in the setting of a peer-supported intervention in which participants are aiming to move through the material as a group. Presenting content in an adaptive fashion might lead to some participants referring to content that others might not have seen. Thus, there is a design tension that must be considered when providing a more personalized experience and a unified group experience that members share.

It is useful to consider the multiple factors that may be at play in the perception of pressure to finish the assignments. Lack of motivation, feeling inadequate, and feeling pressured can also be symptoms of depression, which the intervention is intended to address; it may not be in the participants’ interests to cater to their preferences completely. The design of digital mental health technologies involves a competing tension between promoting engagement and achieving improvements in symptoms and well-being. Behavior change often involves confronting problems that can, at times, be uncomfortable. Thus, the goal of addressing the issues raised in this study may be to minimize the pressure experienced by users by allowing meaningful engagement, recognizing that some users may nevertheless continue to experience some discomfort.

A second design takeaway would be to simplify the instructions and training that are provided to the participants. Previous research has also argued that the availability of support is critical as older adults are learning a technology, although this support can take various forms, including step-by-step guidance, an error-friendly space where someone is available to fix problems if they arise, and manuals [14]. Owing to the complexity of the system used in this study, the training materials that the participants received were extensive, and it may have been difficult to find what they needed at the appropriate time. Providing simpler training materials, a short summary of key actions that is accessible from anywhere in the program, and training videos could perhaps enable participants to engage with the site more easily and ameliorate the confusion and frustration experienced during the study. Having these features might also reduce the burden on coaches for providing technical support and training and preventing intervention dropout.

Although disorientation and cognitive overload are well-recognized as concerns in the interface design [42], this qualitative analysis has shown that they take a particular meaning with this population and that addressing these issues is paramount. User interface adaptations that involve the reduction of interface complexity or enhancement of assistance features can be particularly important for older adults to mitigate the effects of cognitive decline and vision loss [43] and prevent normal user interface difficulties from being interpreted as evidence of cognitive decline or lack of competence.

### Limitations and Future Directions

Our analysis had various limitations that could also serve as potentially fruitful directions for subsequent research. First, one of the data types that we analyzed, reframing negative thoughts, were a skill that participants were explicitly directed to practice. As such, these thoughts were inherently negative in nature, and both the life challenges and negative aspects of the participants’ intervention experiences might be more apparent than positive aspects due to our selection of data to analyze. Second, in addition to the quantitative and qualitative analyses that have been conducted, analytical methods that model the participants’ interactions, such as network analysis, could have provided additional insight into how the participants interacted with one another and could be a fruitful direction for future research. Third, the findings are based on a secondary analysis of the content produced during the course of the intervention; we did not directly conduct an exit interview asking the participants to comment on their experiences or specific features such as nudges, which could have led to additional insights to inform the design and features of the intervention. Finally, our sample primarily comprised individuals who were female, white, and highly educated. There is a need to further explore how individuals from other backgrounds might experience this intervention.

### Conclusions

In this paper, we analyzed textual data from an internet intervention with a peer support component for older adults with symptoms of depression. The textual data provided insight into the participants’ experiences with the intervention as well as design considerations for developing complex technological interventions that support the challenges that participants may experience due to aging and cognitive difficulties. First, technical issues encountered by older adults within the context of the intervention can interact with and exacerbate insecurities that they may experience in life, and it is important to consider how intervention components might be designed to mitigate these issues. Second, peer support can be employed as a mechanism to facilitate communication, support, and collaborative problem solving among participants in an intervention. It is our hope that insights from this study inform the design of other iCBT interventions for older adults.

## Abbreviations

CBT: cognitive behavioral therapy
iCBT: internet-based cognitive behavioral therapy
IPA: interpretative phenomenological analysis
